# Uropathogenic Pan-Drug-Resistant Providencia rettgeri Infection in a Patient With an Indwelling Urinary Catheter: A Case Report

**DOI:** 10.7759/cureus.73318

**Published:** 2024-11-09

**Authors:** Mahadevaiah Neelambike Sumana, Yogeesh D Maheshwarappa, Morubagal Raghavendra Rao, Deepashree R, Krishna Karthik MVS, Nikita K Shah

**Affiliations:** 1 Microbiology, JSS Medical College and Hospital, JSS Academy of Higher Education and Research, Mysuru, IND; 2 Clinical Microbiology, JSS Medical College and Hospital, JSS Academy of Higher Education and Research, Mysuru, IND

**Keywords:** catheter-associated urinary tract infection, indwelling urinary catheter, multi-drug-resistant organisms, nosocomial infection, providencia rettgeri

## Abstract

This case report details the clinical management and implications of infection with pan-drug-resistant *Providencia rettgeri* in a 50-year-old male admitted and diagnosed with acute peritonitis due to hollow viscus perforation, highlighting an emerging challenge in healthcare settings. Following emergency laparotomy and intensive care admission, the patient was catheterized to assist urine drainage and subsequent urine bacterial culture which yielded pan-drug-resistant *P. rettgeri*, signifying a notable instance of nosocomial infection by a multi-drug-resistant organism. Despite the organism’s resistance to broad-spectrum antibiotics, clinical improvement was observed with levofloxacin treatment, underlining the potential discrepancy between in vitro resistance patterns and in vivo response, particularly in urinary tract infections (UTIs) where urine drug concentrations are pivotal. This case underscores the necessity for heightened surveillance, stringent infection control measures, and judicious antibiotic use in managing infections caused by pan-drug-resistant pathogens like *P. rettgeri*, challenging the traditional paradigms of antimicrobial resistance and therapy in hospital settings.

## Introduction

*Providencia rettgeri* is a motile, gram-negative bacillus belonging to the Enterobacteriaceae family. The different species of the genus Providencia include *Providencia alcalifaciens*, *Providencia rustigianii*, *Providencia stuartii*, *P. rettgeri*, and *Providencia heimbachae*. It is commonly found in soil, water, and sewage [1,2]. Providencia is an opportunistic human pathogen commonly associated with urinary tract infections (UTIs), gastroenteritis, and septicemia in immunocompromised individuals [3]. It is also increasingly reported in cases of pneumonia, neonatal septicemia, and burns. Additionally, Providencia has the potential to cause nosocomial infections, particularly catheter-associated urinary tract infections (CAUTI), especially with long-term indwelling urinary catheters in the elderly population [3-5]. Patients with *P. rettgeri* infection are also known for developing urinary tract stones, long-term urinary catheter obstruction, and complicated pyelonephritis [5,6]. The mortality rate is high among the elderly population when Providencia species are associated with UTIs or bacteremia. To our knowledge, we are reporting the first case of pan-drug-resistant nosocomial uropathogenic *P. rettgeri* isolated from an elderly patient with an indwelling urinary catheter, particularly in this region (Karnataka, South India).

## Case presentation

A 50-year-old male with no travel history and no known comorbidities having acute onset of severe upper abdominal pain accompanied by diffuse abdominal distension was brought to the Emergency Department of JSS Hospital, Mysuru, Karnataka. The patient had a history of smoking for 20 years and alcohol intake for 25 years, with alcohol consumption reported on the day before the onset of symptoms. Upon physical examination, the patient was afebrile, conscious, alert, and oriented to time, place, and person. No signs of pallor, icterus, cyanosis, clubbing, pedal edema, or lymphadenopathy were observed. The vital signs were within normal limits with a pulse rate of 98 beats per minute, a respiratory rate of 18 per minute, and an oxygen saturation of 98% at room air. He was hypotensive with the blood pressure measuring 80/60 mmHg.

**Figure 1:**
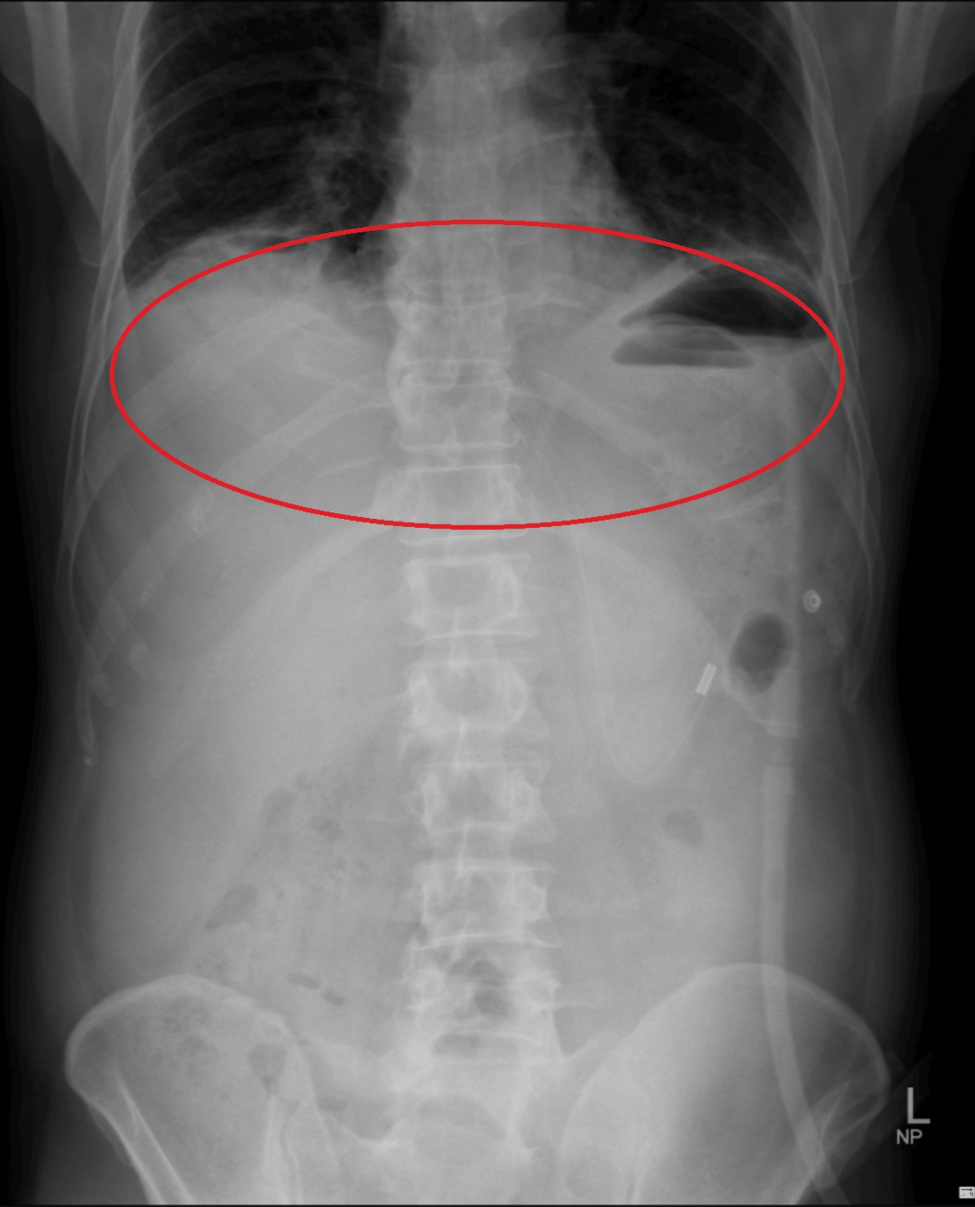
Abdomen X-ray showing air under diaphragm The marked area shows air under the diaphragm

Systemic examination revealed no abnormalities except for a mild abdominal distension with guarding and epigastric tenderness. A radiological investigation of the abdomen revealed the presence of air under the diaphragm, as shown in Figure 1.

Based on these findings, the patient was diagnosed with peritonitis secondary to hollow viscus perforation. Immediate treatment included initiation of inotropic support and emergency laparotomy, during which Graham’s omental patch repair was performed for the prepyloric perforation. Post-operatively, the patient was intubated, and a Foley catheter was inserted for further management in the surgical intensive care unit (SICU). Intravenous meropenem (1 mg IV eighth hourly) and metronidazole (500 mg IV eighth hourly) antibiotics, as well as hydrocortisone (100 mg IV once daily), were initiated for the patient.

During the patient's stay in the ICU, on the third post-operative day, he extubated himself and removed the pelvic drain that had been placed. Due to developing an irritable behavioral change, he required psychiatric consultation, for which the appropriate medication was prescribed. Additionally, the patient developed hypoalbuminemia, hypocalcemia, thrombocytopenia, and electrolyte imbalances, which were corrected accordingly. On the 12th postoperative day, an examination of the surgical wound revealed exposure of the bowel. An emergency mass closure of the abdomen under general anesthesia was performed. Subsequently, the patient was started on an intravenous cefoperazone-sulbactam antibiotic after being transferred to the SICU.

As the patient was intubated, routine diagnostic samples were collected from the endotracheal tube and surgical wound site along with blood samples which showed the growth of multi-drug-resistant *Klebsiella pneumoniae*, prompting an escalation of antibiotic therapy to intravenous colistin. A routine urine sample collected due to the presence of a Foley catheter revealed the growth of a pan-drug-resistant strain of *P. rettgeri*. A repeat urine sample collected with thorough aseptic precautions also showed the same growth of pan-drug-resistant *P. rettgeri *(photograph not available). The report was sent detailing *P. rettgeri *along with the antibiotic susceptibility patterns and minimum inhibitory concentration values as shown in Table 1. The isolate was found to be resistant to all drugs by the Vitek-2 Gram-negative bacilli (GNB) 405 and 407 cards which had passed quality control checks. Other laboratory investigation results are detailed in Table 2.

**Table 1:** Antibiotic sensitivity pattern of the isolate - indicates MIC of the antibiotic not given because manual susceptibility testing is followed for the antibiotic testing. MIC: minimum inhibitory concentration

Antibacterial agent	MIC	Interpretation
Amikacin	≥64	Resistant
Cefoxitin	≥64	Resistant
Colistin	≥16	Resistant
Doxycycline	≥16	Resistant
Tetracycline	≥16	Resistant
Tobramycin	≥16	Resistant
Amoxicillin/clavulanic acid	-	Resistant
Ampicillin	-	Resistant
Ampicillin/sulbactam	≥32	Resistant
Cefepime	≥32	Resistant
Ceftazidime/avibactam	≥16	Resistant
Chloramphenicol	≥64	Resistant
Ceftriaxone	-	Resistant
Cotrimoxazole	-	Resistant
Meropenem	≥16	Resistant
Levofloxacin	≥8	Resistant

**Table 2:** Laboratory findings ELISA: enzyme-linked immunosorbent assay; ELFA: enzyme-linked fluorescence assay; HBV: hepatitis B virus; HCV: hepatitis C virus

Physical examination	
Parameter	Observation
Blood pressure	80/60 mmHg
Pulse rate	98 bpm
SPO_2_	98% at RA
Febrile status	Afebrile
Respiratory rate	18/min
Systemic examination	
Cardiovascular system	S1 and S2 +
Respiratory system	Bilateral normal vesicular breath sounds
Per-abdomen examination	Abdominal distension present Epigastric tenderness present Voluntary guarding present Umbilicus central and inverted No other abnormalities
Central Nervous system	Conscious, oriented, no focal neurological deficit
Radiological findings	
X-ray - erect abdomen	Air under the diaphragm
Laboratory investigations	
serum glutamic oxaloacetic transaminase (SGOT)	71 U/L
Serum glutamic pyruvic transaminase (SGPT)	48 U/L
Total bilirubin	1.14 mg/dl
Direct bilirubin	0.56 mg/dl
Total proteins	5 gm/dl
Serum albumin	2.5 gm/dl
Hemoglobin	9.4 gm/dl
Hematocrit	35.5%
Serum calcium	7.2 mg/dl
D dimer	8.7 microgram FEU/ML
Serological findings	
HIV	Negative
HBV	Negative
HCV	Negative
Dengue NS1 antigen detection (ELFA)	Negative
Dengue IgM antibody detection (ELFA)	Negative
Weil Felix test	Negative (titer less than 1 in 80)
Leptospira Ig M ELISA	Negative
WIDAL test	Negative
Procalcitonin	5.71 ng/ml 7.78 ng/ml 0.29 ng/ml

The patient's condition appeared to be improving, as he tolerated oral feeds and was successfully tapered off and discontinued from inotropic support. Consequently, he was transferred to the step-down SICU. The pelvic drain and subhepatic drain that had been placed were gradually removed. Vacuum-assisted closure (VAC) dressing was applied upon examination of the surgical wound and secondary suturing was performed. Given the deranged liver function tests, a medical gastroenterology opinion was sought. On request, the patient was shifted to the general ward under high-risk consent against medical advice. On the 28th postoperative day, the patient experienced hypotension and desaturation, necessitating the re-initiation of inotropic support. The patient and his attendees were strongly advised to transfer him back to the ICU for further care. However, they refused further treatment leading to the patient's discharge against medical advice. After discharge, the patient was admitted to the district government hospital where the consultant replaced the Foley's catheter and a fresh urine sample was sent for culture. The culture isolated the same pan-drug-resistant *P. rettgeri* with the identical antibiogram as detailed in Table 1. The patient was initiated on an injection of levofloxacin (500 mg IV once daily). Symptomatically, the patient began to improve and was discharged upon completing the antibiotic course.

## Discussion

The case presented here highlights the emergence of *P. rettgeri *as a significant nosocomial pathogen, particularly among catheterized patients in our geographical area (Mysuru, Karnataka). Although relatively rare,*Providencia *species are quite common in the environment and have been known to cause outbreaks [5-7]. In this instance, the isolated strain of pan-drug-resistant *P. rettgeri* further emphasizes the challenge posed by multi-drug-resistant pathogens in healthcare settings.

*P. rettgeri* shares characteristics with other members of the Enterobacteriaceae family, such as *Proteus *and *Morganella*. *Providencia* is a pathogen that can cause a range of infections including urinary tract, respiratory tract, gastrointestinal tract, skin and soft tissue infections, and septicemia, underscoring its clinical significance [7]. Moreover, its association with immunocompromised individuals including those with prolonged catheterization, highlights the need for heightened vigilance in healthcare settings by increasing the surveillance measures.

Previous reports have documented outbreaks of *Providencia* species. In 1981, Fierer and Ekstrom reported a small outbreak of *Providencia *species in condom-catheterized patients at the Veterans Administration Medical Center, San Diago [8]. In 2016, Tshisevhe et al. reported a small outbreak of carbapenem-resistant *P. rettgeri* in immunocompromised patients of Steve Biko Academic Hospital, Protea, South Africa [9]. This indicates its potential for nosocomial transmission. Long-term catheterization has been identified as a risk factor for *Providencia* infections, as evidenced by studies conducted by Akbaş​​​​​​ et al. (1995) and Tshisevhe et al. (2016) [10]. This observation aligns with the case presented here, where the patient's indwelling urinary catheter likely facilitated colonization and subsequent infection with pan-drug-resistant *P. rettgeri*.

In the Indian context, the emergence of multi-drug-resistant *P. rettgeri* has been documented in recent years. Cases reported by Sagar et al. in 2016 from Manipal, Karnataka, and Mahajan et al. in 2022 from South India highlight the clinical significance of this pathogen, particularly among catheterized patients [4,7]. The case presented here adds to the existing literature, representing the third reported case from Karnataka and emphasizing the need for good infection control practices, continued surveillance, and management strategies tailored to address multi-drug-resistant *Providencia *infections.

The observed symptomatic improvement in the patient following levofloxacin treatment may be attributed to various factors, including the pharmacokinetic properties of quinolones and their ability to achieve higher concentrations in urine. Quinolones, including levofloxacin, are known for their excellent urinary excretion and concentration, making them particularly effective against urinary tract pathogens including *P. rettgeri *[5,6]. Furthermore, the discrepancy between in vitro resistance patterns and in vivo treatment responses underscores the importance of considering factors beyond in vitro antimicrobial susceptibility testing alone. Levofloxacin's broad-spectrum coverage against gram-negative bacteria, coupled with its favorable pharmacokinetic profile, likely contributed to the patient's clinical recovery by effectively targeting the underlying infection. This case highlights the significance of understanding both the pharmacodynamic and pharmacokinetic properties of antibiotics in optimizing treatment outcomes, especially in the context of multi-drug-resistant/pan-drug-resistant infections.

## Conclusions

Apart from *Enterococci, Staphylococcus, Klebsiella, Acinetobacter, Pseudomonas, Escherichia coli,* and *Enterobacter* (ESKAPE) pathogens, which are known to be multi-drug-resistant, extensively drug-resistant, or pan-drug-resistant, we also need to keep in mind the possibility of infection with other gram-negative bacilli in managing the drug-resistant infections. This patient responded to levofloxacin probably because of the increased concentration of quinolone excreted in the urine, and levofloxacin is known to be a urinary quinolone. The discrepancy between in vitro and in vivo responses is already known. In this kind of situation, stringent hospital infection control practices need to be implemented such that rare drug-resistant pathogens are contained without having to include them among ESKAPE pathogens. One needs to be vigilant of pan-drug-resistant organisms. All possible efforts should be made to salvage the available antibiotics for posterity.
